# Diagnostic and Therapeutic Challenges With Synchronous Lung Adenocarcinoma and Cervical Carcinoma: A Case Report

**DOI:** 10.7759/cureus.94352

**Published:** 2025-10-11

**Authors:** Amitabh Kumar Upadhyay, Manoj Kumar, Aaditya Prakash, Abhishek Kumar, Radhika Narayan

**Affiliations:** 1 Medical Oncology, Tata Main Hospital, Jamshedpur, IND; 2 Radiation Oncology, Tata Main Hospital, Jamshedpur, IND; 3 Nuclear Medicine, Tata Main Hospital, Jamshedpur, IND; 4 Pathology, Tata Main Hospital, Jamshedpur, IND

**Keywords:** cervical carcinoma, lung adenocarcinoma, rare, smpc, synchronous

## Abstract

Cervical cancer and lung cancer are both very rampant cancers and contribute significantly to the global healthcare burden. We discuss the rare occurrence of synchronous multiple primary cancer (SMPC) in an elderly female patient with squamous cell carcinoma (SCC) cervix and lung adenocarcinoma.

The patient had SCC in cervical biopsy, and poorly differentiated carcinoma was reported in the biopsy of a single lung nodule, which was considered metastatic initially.

She received a palliative intent chemotherapy with paclitaxel and carboplatin. Chemotherapy led to a complete response in the cervix, but the lung lesion persisted. Immunohistochemistry on the lung nodule was done, and it guided us to a SMPC diagnosis with lung adenocarcinoma plus SCC cervix. Subsequently, the patient received stereotactic body radiation therapy for lung adenocarcinoma and chemo-radiation for the SCC cervix, and she is on regular follow-up.

The article highlights the practical difficulties in diagnosing SMPC in real-world situations with various practical and financial constraints. The manuscript discusses challenges in defining an optimal treatment regimen for SMPC due to its infrequency, the significance of clinical acuity, and the need for a high index of suspicion. Clinicians must not presume every non-regional nodule is metastatic, especially in cases with an oligometastatic disease burden.

## Introduction

Cervical cancer, which is predominantly squamous cell carcinoma (SCC), is the fourth most common cancer among women globally, following breast, colorectal, and lung cancers [1,2]. In 2020, there were an estimated 604,127 cases of cervical cancer, leading to 341,831 deaths [1,2]. The persistent infection of high-risk human papillomavirus (HPV) is recognized as the primary cause of this disease [3]. Alarmingly, approximately 85% of cervical cancer deaths occur in underdeveloped or developing countries, where the mortality rate is 18 times higher than in affluent nations [4]. 

Lung cancer is the second most commonly diagnosed cancer worldwide, following breast cancer. It primarily affects men and accounts for approximately 2.2 million cases and 1.8 million deaths each year [2,5,6]. According to the 2021 WHO classification [6], the three most prevalent types of lung cancer are adenocarcinoma, SCC, and neuroendocrine cancers. Adenocarcinomas often show positive results for targetable driver mutations, such as those in the epidermal growth factor receptor, anaplastic lymphoma kinase (ALK), B-RAF, and ROS1 genes [7,8]. However, effective screening methods have detected many asymptomatic high-risk patients, particularly in developed countries [9].

Synchronous multiple primary cancer (SMPC) is a sporadic occurrence where two malignant neoplasms arise simultaneously or within six months of each other [10]. This is distinct from metachronous cancers, which occur sequentially with more than six months of separation. The diagnosis of SMPC is based on the criteria outlined by Warren and Gates, which require that both neoplasms must be malignant, anatomically separate, and exclude the possibility of the second primary neoplasm being a metastasis from the index tumor [10].

Here, we present a distinctive SMPC case comprising SCC of the cervix and lung adenocarcinoma in an elderly female patient. The main dilemma is determining the optimal order of diagnostic and therapeutic interventions, especially when financial limitations prevent desired investigations. The text also discusses the practical challenges clinicians face in making treatment decisions at a high-volume center, where significant reliance on clinical judgment is required due to limited resources.

## Case presentation

A 64-year-old woman, housewife by occupation, presented with complaints of postmenopausal vaginal bleeding for two months. She was a non-alcoholic, non-smoker, and had hypothyroidism controlled with medications. She had an Eastern Cooperative Oncology Group (ECOG) performance status of one, was conscious and oriented, and had no pallor, icterus, or edema. Upon external visual inspection, a leukoplakic patch was found over the labia minora. On speculum and vaginal examination, the uterus was normal in size and mobile; the fornices were free, and the cervix was replaced by approximately four-centimeter-sized cauliflower-like growth. On rectal examination, the rectal mucosa was free. Her obstetric history was Gravida 5, Parity 5, Living 5, and Abortion 0 (G5P5L5A0) with all normal vaginal deliveries. 

**Figure 1:**
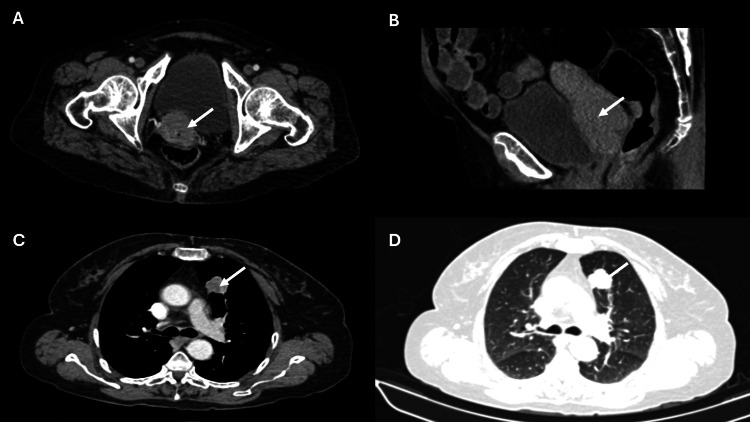
Cross-sectional (A) and sagittal (B) CT images reveal enhancing soft tissue lesions involving the cervix (white arrows). Axial images (C & D) reveal enhancing nodular soft tissue lesions in the anterior segment of the left lung upper lobe (white arrows).

**Figure 2:**
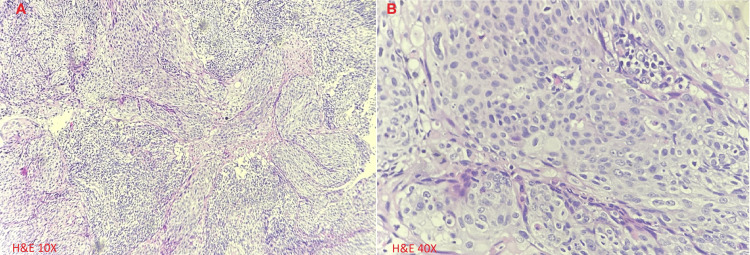
A: Cervix biopsy (Hematoxylin and Eosin x 10) shows invading islands of moderately differentiated non-keratinizing squamous cell carcinoma with moderate peritumoral lymphocytic infiltration; B: Nests of non-keratinizing squamous cell carcinoma (Hematoxylin and Eosin x 40).

**Figure 3:**
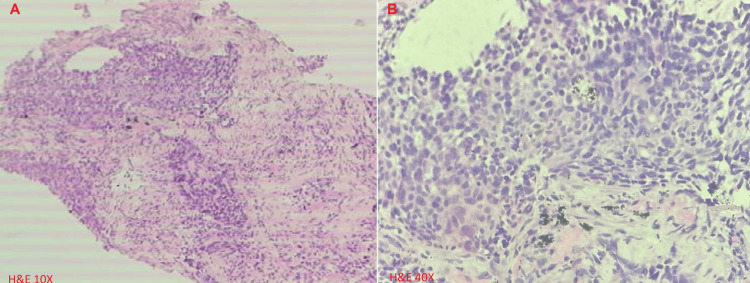
Lung biopsy tissue showing discohesive groups of tumor cells consistent with non-keratinizing poorly differentiated carcinoma, A: In low-power view (Hematoxylin and Eosin x 10); B: In high-power view (Hematoxylin and Eosin x 40).

She has normal liver, kidney, and cardiac functions. Her workup is summarized in Table 1. Her contrast-enhanced computed tomography (CECT) of the abdomen, pelvis, and chest findings are shown in Figure 1. Cervical biopsy is shown in Figure 2, and lung biopsy in Figure 3. Due to economic constraints, we could not perform upfront PET CT, MRI, and immunohistochemistry (IHC) on the cervical and lung tissues to prove HPV association and confirm histology. The unaffordability of the desired investigations was discussed and documented in the records for medicolegal purposes. 

**Table 1:** Baseline parameters

Investigation	Findings
CECT abdomen and pelvis	There is a 4.3 CM x 4.10 CM x 3.6 CM mass in the cervix region with irregular margins. The fat planes between this mass and the bladder were lost with the involvement of the base of the bladder.
CECT thorax	A well-defined lesion measured approximately 2.6 CM × 2.2 CM× 2.4 CM in the anterior segment of the left lung upper lobe. It was of uniform density with contrast enhancement, suspicious of metastasis. No pleural thickening and effusion or abnormal mediastinal lymph nodes were seen.
Cervical biopsy	Moderately differentiated, non-keratinizing squamous cell carcinoma.
Cystoscopic biopsy	The unhealthy area at the trigone of the urinary bladder revealed no evidence of malignancy.
Lung biopsy	Metastatic, poorly differentiated carcinoma.

The case was discussed in our institutional multidisciplinary board, and the differential diagnosis of oligometastatic SCC cervix vs. the SMPC was discussed in detail. Based on the radiological, pathological, clinical history, and the unaffordability for the desired investigations, it was decided to start with chemotherapy first and monitor the biological behavior of the disease, prior to offering any definitive treatment. 

**Figure 4:**
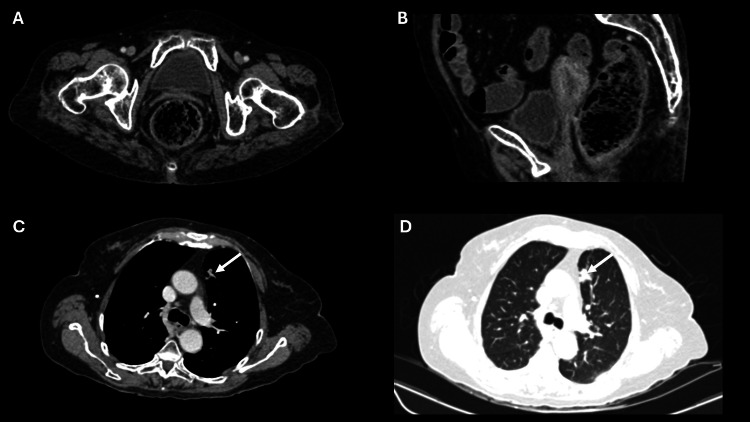
Cross-sectional (A) and sagittal (B) CT images reveal the resolution of the lesion involving the cervix. Cross-sectional images (C & D) reveal a persistent enhancing nodular soft tissue lesion in the anterior segment of the left lung upper lobe (white arrows).

**Figure 5:**
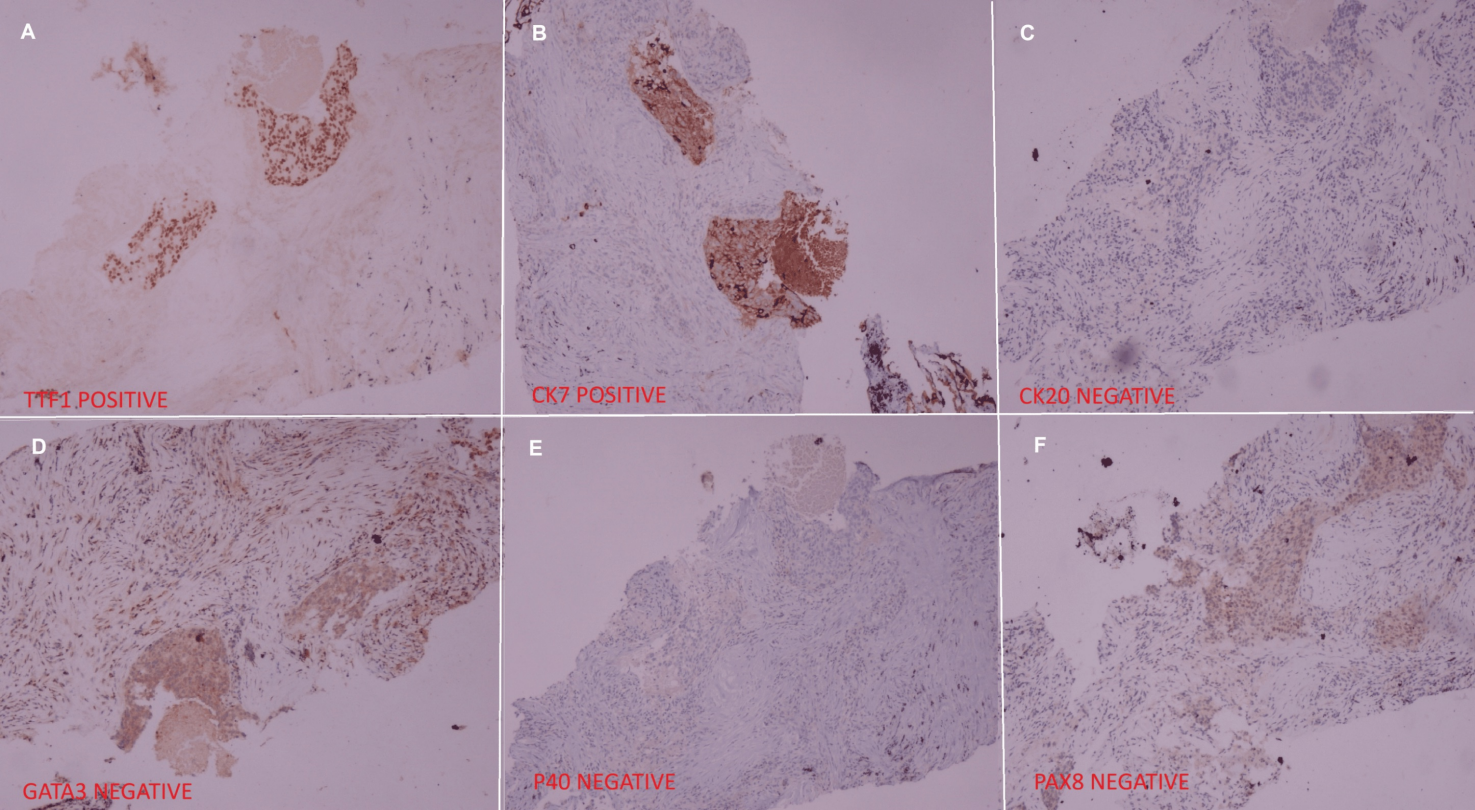
Immunohistochemistry images showing positive staining for TTF1 (A) and CK7 (B), while negative staining for CK20 (C), GATA-3 (D), P40 (E), and PAX8 (F), suggestive of adenocarcinoma of pulmonary origin.

Initially, the patient received systemic therapy with four cycles of three weekly paclitaxel and carboplatin. Bevacizumab or pembrolizumab could not be added due to financial constraints. Post-four cycles of chemotherapy response assessment revealed a 15 mm x 11 mm speculated enhancing nodular lesion in the lung. There was no residual disease in the cervical region, enlarged pelvic nodes, or ascites. Overall, the CECT findings suggested a good partial response (Figure 4). Given the persistence of the lung lesion and the absence of residual disease in the cervix, left lung mass immunohistochemistry (IHC) was done after counselling, which revealed a positive TTF1 (4+) and CK-7 (3+). At the same time, CK20, GATA-3, PAX-8, and P40 were non-immunoreactive in lesional cells (Figure 5). Overall, IHC was reported as non-small cell carcinoma, favoring adenocarcinoma of the lung. Following the diagnosis of lung adenocarcinoma, a CECT brain was recommended, and it showed no signs of any metastatic lesion.

The case was re-discussed in our institutional multidisciplinary board, with the updates about the IHC and imaging, and a diagnosis of cervical carcinoma (FIGO stage IIA) with adenocarcinoma lung (T1cN0M0) was made. The patient was planned for SBRT for lung carcinoma and definitive chemotherapy plus radiation therapy for the cervical carcinoma. The patient was counseled for the surgery for the lung lesion, but she denied due to her personal reasons. In the first phase, she received SBRT to the lung nodule in a 50 Gray (Gy) dose for five fractions in an alternate-day schedule. She received intensity-modulated radiation therapy to the cervix in a dose of 50 Gy for 25 fractions with concurrent chemotherapy with cisplatin in a dose of 40 mg/m^2^ once weekly schedule for five weeks, followed by weekly intra-cavitary brachytherapy with 21 Gy in three sessions with 7 Gy in each session. 

**Figure 6:**
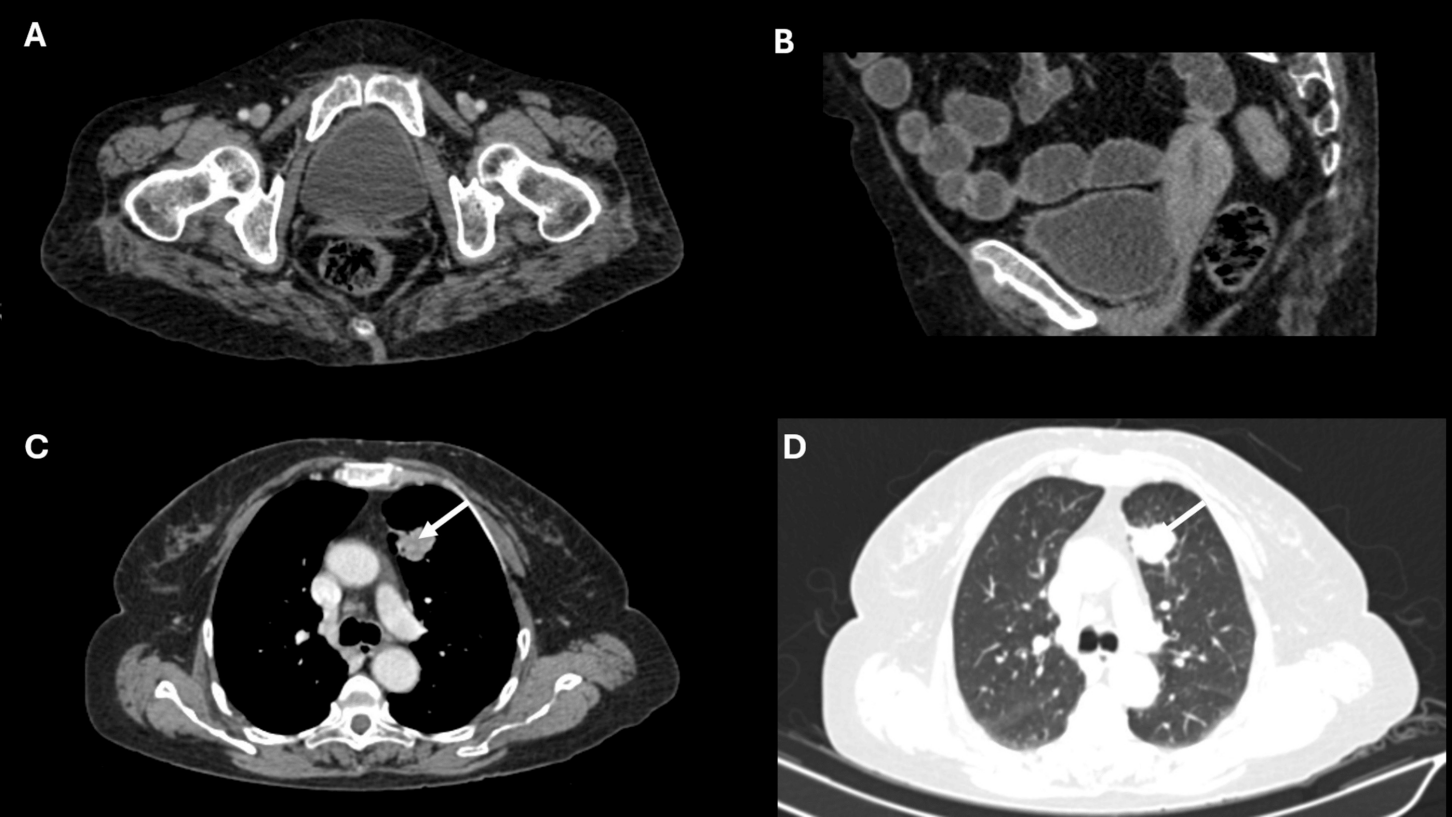
Cross-sectional (A) and sagittal (B) CT images reveal no evidence of enhancing lesions involving the cervix. Cross-sectional images (C & D) reveal fibroreticular opacities in the region of the previously seen lesion in the anterior segment of the left lung upper lobe (white arrows).

Six weeks after treatment, CECT revealed approximately 12 x 13 mm-sized irregular inhomogeneous soft tissue lesions in the lung with fibrotic bands and tractional bronchiectasis. The cervix appeared normal in shape and size without any focal detectable mass in the uterus, cervix, or pelvis (Figure 6). The patient is asymptomatic and has been on regular follow-up for the last 15 months with clinical examinations, liver function tests, kidney function tests, hemogram at three-month intervals, and CECT thorax and whole abdomen, at every six-month interval. 

## Discussion

SMPC is a rare occurrence where two malignant neoplasms arise simultaneously or within six months of each other [10]. The diagnosis of SMPC is based on the criteria outlined by Warren and Gates, which require that both neoplasms must be malignant, anatomically separate, and exclude the possibility of the second primary neoplasm being a metastasis from the index tumor [10]. The diagnosis and management of SMPC present a considerable challenge to clinicians.

There is a paucity of literature regarding analogous instances, and our case represents probably the third instance of SMPC with a combination of cervical carcinoma and lung adenocarcinoma. The occurrence of four primary cancers in a single patient has been reported, which included synchronous cervical carcinoma, lung carcinoma, metachronous basal cell carcinoma of the skin, and rectal carcinoma [11]. Treatment sequencing is not available for this patient. Another case with a history of heavy smoking with synchronous advanced cervical cancer and metastatic NSCLC was reported in a 48-year-old female patient. Her NSCLC showed no actionable markers, and she received paclitaxel and cisplatin as palliative chemotherapy to start with [12]. She responded well in the lung part, but the cervical lesion progressed, necessitating palliative intent surgery and then further chemotherapy and immunotherapy with nivolumab. Our case is different in clinical behavior, where she had a complete response in the cervical part and stable disease in the lung part. Moreover, the lung lesion was nonmetastatic in our patient, so the molecular profiling was not done. We chose a paclitaxel and carboplatin regimen, which was likely to benefit both cancers. Bevacizumab and pembrolizumab were valid options for cervical cancer, but affordability was a concern. 

The treatment of non-metastatic cervical carcinoma typically involves surgery in early cases and definitive chemo-radiation and brachytherapy in more advanced diseases [13]. Although the biopsy of the lung nodule showed a poorly differentiated carcinoma, the patient was initially treated with palliative chemotherapy, under the impression of SCC cervix with lung metastasis. Initially, a synchronous double malignancy was not assumed due to its sporadic occurrence. In real-world scenarios, clinicians have to deal with many such situations where all desired investigations cannot be done due to many constraints. Our case was not affordable for PET scan/MRI/IHC/molecular testing/bevacizumab and was managed accordingly after discussing and documenting the ethical and medicolegal concerns. The affordability of cancer treatment is a huge concern across the world, with the exclusion of some developed nations [14]. The concerns related to cancer treatment in India and other low- and middle-income countries are related to access, affordability, and disruptions in treatment [15]. Most of the time, a clinician practicing at a high-volume center catering to poor patients is forced to amend ideal staging evaluations, treatment plans, response assessments, etc. Disease behavior and subsequent clinical course become vital for decision-making in these situations. A policy by the government may reduce the affordability concerns for high-cost imaging options and molecular testing. Similarly, policy must include the most essential targeted therapy options for cancer cases. The prognosis of the SMPCs is variable, as the diseases are biologically different and the prognositication becomes very challenging for treating oncologists. 

There is the possibility of clinicopathological heterogeneity or phenotype discordance between primary and metastatic sites, which is reported in the literature, and the same possibility was also discussed and assumed [16]. Following chemotherapy, the cervical mass exhibited a complete response, while the lung nodule persisted, indicating a different biology. Upon performing IHC, a diagnosis of SMPC was ultimately reached, highlighting a significant learning point. IHC was positive for TTF and CK-7 and negative for PAX-8 and P40, guiding to a diagnosis of lung adenocarcinoma and, at the same time, ruling out cervical SCC metastasis [17]. 

The patient declined surgical treatment for lung cancer, citing personal reasons and her age. However, surgery was still a viable option at this stage [18]. If this case were diagnosed as SMPC upfront, we could probably have offered her the same chemotherapy with paclitaxel and platinum first, followed by definitive local therapy for both sites. This approach could have shown the biological behavior of the two diseases prior to offering definitive treatment for both sites. Since the optimum treatment sequencing is not defined, clinicians can also choose other strategies, depending upon the performance status and preferences. The other possible approach in a proven case of SMPC with localized cervical and lung adenocarcinoma is upfront chemoradiation for the cervical part and surgery followed by adjuvant chemotherapy for the lung part. We also have the option of neoadjuvant chemotherapy with paclitaxel and carboplatin for the cervix part, followed by definitive chemoradiation, as per the Interlace protocol [19]. The concern for cumulative toxicity is there in SMPC, as platinum is a key drug in most chemotherapy doublets and concurrent regimes. Our case tolerated the chemotherapy and subsequent chemoradiation well. The definitive treatment options for SMPC in the context of cervical SCC and adenocarcinoma of the lung involve definitive chemo-radiation or surgery for the SCC cervix, followed by SBRT or surgery to the lung, or vice-versa [20-22]. There are pros and cons for every permutation and combination of treatment sequencing, as there will be concerns about the cumulative toxicity and possible progression of other lesions, even if one site is responding. The patient has to tolerate the treatment modalities for both cancers in various sequences, which are very much debatable. Quality of life considerations in view of the cumulative toxicities also play a vital role in treatment discussions. After treatment completion, a detailed follow-up and survivorship plan is paramount.

## Conclusions

The optimal treatment regimen and sequencing for SMPC present a significant challenge for clinicians and depend on several factors, including the location and stage of both tumors, symptom burden, aggressiveness of tumors, chemosensitivity, performance status, patients' preference, affordability of the advised treatment options, tolerance to the treatment with cumulative toxicities, and quality of life considerations. A multidisciplinary approach that involves the participation of surgeons, medical oncologists, pathologists, radiologists, and radiation oncologists is typically employed to determine the optimal approach for these sporadic cases. Maintaining a high degree of suspicion when diagnosing synchronous malignancies is crucial. This case underscores the significance of considering rare occurrences and exercising clinical acuity when managing such cases and emphasizes the importance of IHC. In the absence of clear guidelines, this case provides valuable insights for the healthcare community regarding the etiology and management of such infrequent events. More and more reporting of these cases would make our scientific database robust and would help in coming to a consensus for treating these rare cases.
